# *Burkholderia multivorans* Infections Associated with Use of Ice and Water from Ice Machines for Patient Care Activities ― Four Hospitals, California and Colorado, 2020–2024

**DOI:** 10.15585/mmwr.mm7339a4

**Published:** 2024-10-03

**Authors:** Axel A. Vazquez Deida, Kevin B. Spicer, Kiara X. McNamara, Matthew J. Arduino, Paige Gable, Alison L. Halpin, Lindsay J. Caverly, John J. LiPuma, Braden Bardach, Cayla Mayle, Samuel N. Baird, Christopher A. Czaja, Raymond Chinn, Jane D. Siegel, Kiran M. Perkins

**Affiliations:** ^1^Epidemic Intelligence Service, CDC; ^2^Division of Healthcare Quality Promotion, National Center for Emerging and Zoonotic Infectious Diseases, CDC; ^3^University of Michigan, Ann Arbor, Michigan; ^4^Colorado Department of Public Health & Environment; ^5^County of San Diego Health and Human Services Agency, San Diego, California; ^6^California Department of Public Health.

**Summary**

**What is already known about this topic?**

During 2021–2022, contaminated ice and water from ice machines were linked to 23 *Burkholderia multivorans* cases at two southern California hospitals.

**What is added by this report?**

Twenty-three additional, previously unreported *B. multivorans* cases occurred during 2020–2024: 13 at a northern California hospital, eight at a hospital in Colorado, and two additional cases at one of the southern California hospitals. All environmental and clinical isolates were highly genetically similar. The same brand of ice machine and the same filters, descaling, and sanitizing products were used by all four hospitals.

**What are the implications for public health practice?**

During outbreaks of water-related organisms in health care facilities, health care personnel should consider avoiding the use of tap water, including ice and water from ice machines, for patient care.

## Abstract

Ice machines can harbor water-related organisms, and the use of ice or tap water for clinical care activities has been associated with infections in health care settings. During 2021–2022, a total of 23 cases of infection by *Burkholderia multivorans* (sequence type ST659) were reported at two southern California hospitals and linked to contaminated ice and water from ice machines. In addition to these 23 cases, this report also includes 23 previously unreported cases of *B. multivorans* ST659 infections that occurred during 2020–2024: 13 at a northern California hospital, eight at a hospital in Colorado, and two additional cases at one of the southern California hospitals. The same brand of ice machine and brands of filters, descaling, and sanitizing products were used by all four hospitals; *B. multivorans* was isolated from samples collected from ice machines in two of the hospitals. Whole genome sequencing indicated that all clinical and ice machine isolates were highly genetically similar (0–14 single nucleotide variant differences across 81% of the selected reference genome). Recommendations from public health officials to halt the outbreak included avoiding ice and tap water during clinical care activities. An investigation is ongoing to determine possible sources of ice machine contamination. During outbreaks of water-related organisms in health care facilities, health care personnel should consider avoiding the use of tap water, including ice and water from ice machines, for patient care.

## Introduction

Ice machines contain several mechanical components that can favor microbial amplification and biofilm formation (*1*,*2*). Use of ice and tap water for clinical care activities has been recognized as a potential route of transmission of water-related opportunistic pathogens causing health care–associated infections, such as *Burkholderia* spp (*3*,*4*). *Burkholderia multivorans* is a member of the *Burkholderia cepacia* complex, a group of water-related, Gram-negative opportunistic bacteria commonly found in soil and water (*5*). Outbreaks of these organisms have been associated with contaminated medications, aqueous medical products, and medical devices and are of clinical importance because these organisms can be antibiotic-resistant and can lead to severe infections, especially among immunocompromised and critically ill patients (*4*,*6*,*7*). *B. multivorans* is often found in the sputum of patients with cystic fibrosis but is rarely isolated otherwise (*5*,*8*).

During August 2021–July 2022, two hospitals in a health care system in southern California (hospitals B and C) reported *B. multivorans* infections in 23 patients without cystic fibrosis (*9*). The outbreak investigation revealed that ice and water dispensed from ice machines were commonly used for clinical care activities (e.g., bed baths, swallow evaluations, and topical application of ice packs for analgesia). The same sequence type (ST659) of *B. multivorans* identified among the patients was isolated from ice and water samples collected from hospital B ice machines (*9*). This sequence type of *B. multivorans* had not been previously identified in the United States (*10*). Reports of patients without cystic fibrosis with *B. multivorans* infections from other hospitals in California and Colorado prompted CDC and state and local jurisdictions to conduct additional investigations. This activity was reviewed by CDC, deemed not research, and was conducted consistent with applicable federal law and CDC policy.*

## Investigation and Results

### Case Identification

A case was defined as an infection with the outbreak strain (ST659) of *B. multivorans*, documented by isolation from a clinical specimen, in a patient without cystic fibrosis at one of the affected acute care hospitals during January 2020 or afterward. During September 2020–February 2024, a total of 46 cases were identified (Figure). During September 2020–October 2021, 13 cases were identified at a northern California hospital (hospital A). In addition to the 23 cases previously reported at the two southern California hospitals (hospital B = 20 and hospital C = three) during August 2021–July 2022, two additional cases were identified at hospital B during September 2022–February 2023. Eight cases were identified at a hospital in Colorado (hospital D) during April 2023–February 2024. Medical records of all 46 cases were reviewed to identify common exposures.

**FIGURE:**
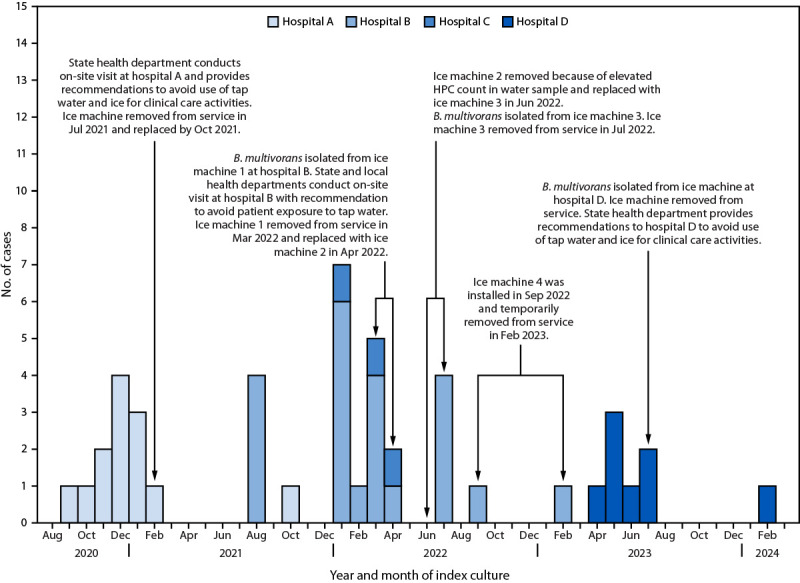
Timeline of the *Burkholderia multivorans* outbreak investigation associated with use of ice and water from ice machines ― four hospitals,* California and Colorado, 2020–2024 **Abbreviation:** HPC = heterotrophic plate count. * Hospital A is located in northern California, hospitals B and C in southern California, and hospital D in Colorado.

Cases in three of the 46 patients were not temporally clustered with other cases: one patient was identified in October 2021 at hospital A, 8 months after the previous case at this hospital; this patient’s previous admission to this hospital was during March–April 2021. The second patient was identified at hospital B in February 2023, 5 months after the previous case at this hospital. The third of these patients was identified at hospital D in February 2024, 7 months after the previous case at this hospital; this patient had multiple admissions to this hospital after undergoing solid organ transplantation in May 2023. These patients might have acquired *B. multivorans* months earlier, during a previous hospital admission.

A respiratory specimen was the source of the *B. multivorans* isolate for most patients identified at the three California hospitals (hospital A = 11 [85%] of 13; hospital B = 17 [77%] of 22; and hospital C = two [67%] of three). At hospital D, however, an intra-abdominal specimen was the isolate source for four of the eight identified patients (Table). Most of the 46 patients were admitted to a medical intensive care unit (ICU) at the time of index specimen collection: hospital A = 13 (100%) of 13; hospital B = 15 (68%) of 22; hospital C = two (67%) of three; and hospital D = five (62%) of eight. Review of medical records did not identify any common exposures.

**TABLE:** Characteristics of patients with *Burkholderia multivorans* ― four hospitals, California and Colorado, 2020–2024

Characteristic	No. (%)				
	California			Colorado	Total N = 46
	Hospital A* n = 13	Hospital B^†^ n = 22	Hospital C^§^ n = 3	Hospital D^¶^ n = 8	
**Age, yrs, median (range) **	60 (42–84)	61 (21–93)	61 (55–97)	56 (34–68)	**60 (21–97)**
**Sex**					
Female	4 (31)	10 (45)	0 (—)	4 (50)	**18 (39)**
Male	9 (69)	12 (55)	3 (100)	4 (50)	**28 (61)**
Source of index culture**					
Respiratory	11 (85)	17 (77)	2 (67)	2 (25)	**32 (70)**
Blood	2 (15)	3 (14)	1 (33)	2 (25)	**8 (17)**
Intra-abdominal^††^	0 (—)	0 (—)	0 (—)	4 (50)	**4 (9)**
Urine	0 (—)	2 (9)	0 (—)	0 (—)	**2 (4)**
**Admission characteristics of index hospitalization^§§^**					
Days from admission to index culture, median (range)	15 (8–22)	14 (0–117)	30 (0–57)	8 (2–17)	**14 (0–117)**
**Patient location at time of specimen collection**					
Medical intensive care unit	13 (100)	15 (68)	2 (67)	5 (62)	**35 (76)**
Medical-surgical ward	0 (—)	7 (32)	1 (33)	0 (—)	**8 (17)**
Other^¶¶^	0 (—)	0 (—)	0 (—)	3 (38)	**3 (7)**
Deceased***	8 (62)	9 (41)	0 (—)	4 (50)	**21 (46)**

* Hospital A was in northern California, and cases were identified during September 2020–October 2021.

^† ^Hospital B was in southern California, and cases were identified during August 2021–February 2023.

^§ ^Hospital C was in southern California, and cases were identified during January–April 2022.

^¶ ^Hospital D cases were identified during April 2023–February 2024.

** Index culture is the first culture in which *Burkholderia multivorans* was isolated. If *B. multivorans* was isolated from blood and nonblood clinical specimens on the same day, the blood isolate was considered as the index culture.

^††^ Includes biliary fluid (two), peritoneal fluid (one), and perihepatic abscess fluid (one).

^§§^ Index hospitalization is the hospitalization that occurs when the index culture is identified and includes the period from admission to discharge. Transmission of *B. multivorans* before the index hospitalization cannot be ruled out for patients with frequent hospital admissions.

^¶¶^ Includes solid organ transplant unit (two) and medical telemetry unit (one).

*** Based on information from medical records; timing and cause of death was not ascertained.

### Infection Prevention and Control Assessments

Public health officials conducted site visits at the affected hospitals at various times after report of the clusters to assess general infection prevention and control practices and identify potential transmission routes for water-related organisms. Health care personnel were interviewed to identify clinical care activities that could have exposed patients to *B. multivorans.* These activities included use of ice and tap water for consumption or clinical care (e.g., placing ice or bags containing ice directly onto surgical incisions or wounds, before percutaneous procedures for analgesia, and when bathing patients with indwelling medical devices without covering and protecting insertion sites). These infection control assessments and health care personnel interviews revealed that 1) patients at all four hospitals might have consumed or been exposed to ice and water dispensed from ice machines during clinical care activities (e.g., swallow evaluations, topical application for analgesia and external cooling, and patient bathing) and 2) the same brand of ice machine and brands of filters, descaling, and sanitizing products were used by all four hospitals.

### Environmental Sampling

Public health officials collected samples from ice machines in patient care areas at all four hospitals. Samples were also collected from sinks located in units where patients had been admitted (e.g., sinks in patient rooms, medication preparation stations, and nutrition rooms) at hospitals A, B, and C. Commonly used aqueous medical products from all three California hospitals underwent laboratory testing (e.g., chlorhexidine mouthwash, ultrasound gel, endoscopic instrument lubricants, and premoistened bathing cloths). Products used to descale and sanitize the ice machines from hospitals B and D were also tested.

**Northern California hospital.** The ice machine from hospital A, located in the unit where the cases were identified, was sampled in March 2021 by a private vendor contracted by the health care facility; *B. multivorans* was not isolated. This machine was removed from service in July 2021 and replaced by October 2021; no additional samples could be collected. *B. multivorans* was not isolated from any other environmental samples or aqueous medical products.

**Southern California hospitals.** At hospital B, *B. multivorans* was isolated in March and July 2022 from ice and water samples and the drain pan of two ice machines located in the ICU (Figure). At hospital C, *B. multivorans* was not isolated from sampled ice machines in July 2022, but a water sample from one of the ice machines had high bacterial concentration, with a heterotrophic plate count^†^ of 102,000 colony forming units/mL, which exceeds the potable water level recommended by the Environmental Protection Agency.^§^ *B. multivorans* was not isolated from any other environmental samples, aqueous medical products, or ice machine descaling and sanitizing products.

**Colorado hospital.** *B. multivorans* was isolated from six of 10 ice machines that were sampled during July 2023–March 2024 at hospital D but was not isolated from the descaling and sanitizing products.

### Genomic Analysis

**Random amplified polymorphic DNA analysis and repetitive extragenic palindromic polymerase chain reaction.** All available clinical (38) and environmental (three) *B. multivorans* isolates from hospitals A, B, and C underwent random amplified polymorphic DNA (RAPD) analysis and repetitive extragenic palindromic polymerase chain reaction (Rep-PCR) testing at the University of Michigan *B. cepacia* Research Laboratory and Repository (BcRLR).^¶^ All 41 isolates from these three California hospitals were genetically closely related (>85% relatedness).

**Whole genome sequencing.** The Colorado State Public Health Laboratory, in collaboration with the University of Michigan BcRLR, performed whole genome sequencing and phylogenetic comparison of *B. multivorans* clinical and environmental isolates from all four hospitals. Clinical isolates from all 46 patients and four environmental isolates (hospital B = three and hospital D = one) from three of the implicated ice machines revealed that all had the same sequence type (ST659) and were all genetically highly similar (0–14 single nucleotide variant differences across 81% of the selected reference genome).**

### Public Health Response

During December 2020–February 2021, state public health officials made infection prevention and control recommendations to hospital A, which included avoiding the use of tap water and ice from ice machines during the clinical care of patients. Public health officials made similar recommendations to hospitals B and C during March–August 2022, and to hospital D in July 2023, focused on reducing the risk for tap water and ice exposure during clinical care activities, especially among vulnerable patients, such as those who are immunocompromised or critically ill. All ice machines identified to have *B. multivorans* were removed from service.

In March 2024, CDC issued a national Epidemic Information Exchange notice to identify additional *B. multivorans* infections. An ongoing investigation is being conducted to determine the possible sources of ice machine contamination.

## Discussion

Epidemiologic and laboratory evidence suggests that contaminated ice and water dispensed from ice machines, all the same brand, were likely sources of exposure among patients identified with *B. multivorans* infections in four hospitals in California and Colorado during September 2020–February 2024. Genomic relatedness of the clinical and environmental *B. multivorans* isolates and identification of the outbreak strain in the same brand of ice machine raises the possibility of a contaminated ice machine component or associated product (e.g., descaling and sanitizing products) leading to contamination of the dispensed ice and water. Differences in the proportion of specimen sources with *B. multivorans* across hospitals suggest various routes of exposure and transmission. For example, the higher proportion of intra-abdominal specimens with *B. multivorans* at hospital D is consistent with the observed practice at this hospital of directly applying refillable ice bags to abdominal surgical wounds and device insertion sites. High bacterial counts, such as that found in water samples from an ice machine at Hospital C, might also indicate risk of transmission of water-associated bacteria to patients.

### Limitations

The findings in this report are subject to at least two limitations. First, although *B. multivorans* was not isolated from samples obtained from descaling and sanitizing products used with the ice machines, the tested products might not have represented the same lot numbers as those used at the time of potential patient exposures. Second, specific clinical exposures to tap water and ice are not documented in patient medical records; therefore, it was difficult to confirm when and how patients could have been exposed to ice and water from the contaminated ice machines.

### Implications for Public Health Practice

To limit the growth and spread of water-related organisms in water distribution systems, including ice machines, health care facilities should devise and implement a water management program and identify all potential pathways of water transmission to minimize patients’ risks for infection (*4*).^††^ During outbreaks of water-related organisms in health care facilities, these facilities should consider avoiding the use of tap water, including ice and water dispensed from ice machines, for patient care. Health care personnel should notify public health officials of health care–associated outbreaks of *B. multivorans*.

## Footnotes

* 45 C.F.R. part 46.102(l)(2), 21 C.F.R. part 56; 42 U.S.C. Sect. 241(d); 5 U.S.C. Sect. 552a; 44 U.S.C. Sect. 3501 et seq.

† A measure of the microbial quality of water.

§
https://www.epa.gov/ground-water-and-drinking-water/national-primary-drinking-water-regulations

¶ Methods for RAPD analysis and Rep-PCR as described in https://doi.org/10.1128/jcm.40.9.3300-3307.2002.

** The data for the *B. multivorans* isolates have been deposited in the National Center for Biotechnology Information BioProject database with links to BioProject accession numbers PRJNA288601 (https://ncbi.nlm.nih.gov/bioproject/288601), PRJNA991232 (https://ncbi.nlm.nih.gov/bioproject/991232), and PRJNA720893 (https://ncbi.nlm.nih.gov/bioproject/720893).

††
https://www.cdc.gov/healthcare-associated-infections/php/toolkit/water-management.html

## References

[1] Cazals M, Bédard E, Soucy C, Savard P, Prévost M. How clean is your ice machine? Revealing microbial amplification and presence of opportunistic pathogens in hospital ice-water machines. J Hosp Infect 2023;141:9–16. 10.1016/j.jhin.2023.08.007 37604277

[2] Kanwar A, Cadnum JL, Xu D, Jencson AL, Donskey CJ. Hiding in plain sight: contaminated ice machines are a potential source for dissemination of Gram-negative bacteria and *Candida* species in healthcare facilities. Infect Control Hosp Epidemiol 2018;39:253–8. 10.1017/ice.2017.321 29382408

[3] Lucero CA, Cohen AL, Trevino I, Outbreak of *Burkholderia cepacia complex* among ventilated pediatric patients linked to hospital sinks. Am J Infect Control 2011;39:775–8. 10.1016/j.ajic.2010.12.005 21664002

[4] Perkins KM, Reddy SC, Fagan R, Arduino MJ, Perz JF. Investigation of healthcare infection risks from water-related organisms: summary of CDC consultations, 2014–2017. Infect Control Hosp Epidemiol 2019;40:621–6. 10.1017/ice.2019.60 30942147 PMC7883772

[5] LiPuma JJ. Update on the *Burkholderia cepacia* complex. Curr Opin Pulm Med 2005;11:528–33. 10.1097/01.mcp.0000181475.85187.ed 16217180

[6] Tavares M, Kozak M, Balola A, Sá-Correia I. *Burkholderia cepacia* complex bacteria: a feared contamination risk in water-based pharmaceutical products. Clin Microbiol Rev 2020;33:e00139-19. 10.1128/CMR.00139-19 32295766 PMC7194853

[7] Shaban RZ, Sotomayor-Castillo C, Nahidi S, Global burden, point sources, and outbreak management of healthcare-associated *Burkholderia cepacia* infections: an integrative review. Infect Control Hosp Epidemiol 2020;41:777–83. 10.1017/ice.2020.184 32441235

[8] Kenna DTD, Lilley D, Coward A, Prevalence of *Burkholderia* species, including members of *Burkholderia cepacia* complex, among UK cystic and non-cystic fibrosis patients. J Med Microbiol 2017;66:490–501. 10.1099/jmm.0.000458 28463663

[9] McNamara K, Wilson WW, Solanky D, Outbreak of *Burkholderia multivorans* among patients at two acute-care hospitals in California, August 2021–July 2022. Antimicrob Steward Healthc Epidemiol 2023;3(Suppl 2):s89–90. 10.1017/ash.2023.353

[10] Baldwin A, Mahenthiralingam E, Drevinek P, Elucidating global epidemiology of *Burkholderia multivorans* in cases of cystic fibrosis by multilocus sequence typing. J Clin Microbiol 2008;46:290–5. 10.1128/JCM.01818-07 18032622 PMC2224285

